# *CD274/PD-L1* gene amplification and PD-L1 protein expression are common events in squamous cell carcinoma of the oral cavity

**DOI:** 10.18632/oncotarget.7593

**Published:** 2016-02-22

**Authors:** Melanie Straub, Enken Drecoll, Nicole Pfarr, Wilko Weichert, Rupert Langer, Alexander Hapfelmeier, Carolin Götz, Klaus-Dietrich Wolff, Andreas Kolk, Katja Specht

**Affiliations:** ^1^ Institute of Pathology, Technical University of Munich, Munich, Germany; ^2^ Institute of Pathology, University of Bern, Bern, Switzerland; ^3^ Institute of Medical Statistics and Epidemiology, Technical University of Munich, Munich, Germany; ^4^ Department of Oral and Maxillofacial Surgery, Klinikum rechts der Isar, Technical University of Munich, Munich, Germany; ^5^ National Center of Tumor Diseases (NCT), Heidelberg, Germany; ^6^ German Cancer Consortium (DKTK), Heidelberg, Germany

**Keywords:** PD-L1, PD-1, oral squamous cell carcinoma, fluorescence in situ hybridization, gene amplification, Pathology Section

## Abstract

Immunomodulatory therapies, targeting the immune checkpoint receptor-ligand complex PD-1/PD-L1 have shown promising results in early phase clinical trials in solid malignancies, including carcinomas of the head and neck. In this context, PD-L1 protein expression has been proposed as a potentially valuable predictive marker. In the present study, expression of PD-L1 and PD-1 was evaluated by immunohistochemistry in 80 patients with predominantly HPV-negative oral squamous cell carcinomas and associated nodal metastasis. In addition, *CD274/PD-L1* gene copy number status was assessed by fluorescence *in situ* hybridization analysis. PD-L1 expression was detected in 36/80 (45%) cases and concordance of PD-L1 expression in primary tumor and corresponding nodal metastasis was present in only 20/28 (72%) cases. PD-1 expression was found in tumor-infiltrating lymphocytes (TILs) but not in tumor cells. *CD274/PD-L1* gene amplification was detected in 19% of cases, with high level PD-L1 amplification present in 12/80 (15%), and low level amplification in 3/80 (4%). Interestingly, *CD274/PD-L1* gene amplification was associated with positive *PD-L1* immunostaining in only 73% of cases. *PD-L1* copy number status was concordant in primary tumor and associated metastases. Clinically, PD-L1 tumor immunopositivity was associated with a higher risk for nodal metastasis at diagnosis, overall tumor related death und recurrence. Based on our findings we propose to include PD-L1 copy number status in addition to protein status in screening programs for future clinical trials with immunotherapeutic strategies targeting the PD-1/PD-L1 axis.

## INTRODUCTION

Squamous cell carcinomas comprise over 95% of malignant tumors in the head and neck region, including the oral cavity. The disease is associated with a poor prognosis, with an overall 5-year survival rate of 60%. Two-thirds of the tumors present with locally advanced or metastatic disease (stages III and IV). Historically, oral cavity squamous cell carcinoma (OCSCC) was viewed as a tobacco- and alcohol-related cancer, but infection with human papillomaviruses (HPVs) has emerged as an important risk factor for OCSCCs (reviewed in [1]) Treatment recommendations according to the National Comprehensive Cancer Network include surgery for patients with early-stage tumors and surgery or definitive concurrent chemoradiotherapy for those with advanced-staged tumors [2].

Tumor immunotherapy targeting the immune regulatory molecules programmed death 1 (PD-1) and its ligand programmed death-ligand 1 (PD-L1) have shown antitumor effects in a subset of patients with solid tumors and have become an interesting prospect for the treatment of head and neck squamous cell carcinoma (HNSCC). HNSCC is an immunosuppressive disease, with tumor cells evading the host immune system through a number of mechanisms, including alteration of their own immunogenicity and production of immunosuppressive mediators (reviewed in [3]). The PD-1/PD-L1 pathway is important for tumor immune escape and promotion of the tumor microenvironment. PD-L1 is a 290aa type I transmembrane surface glycoprotein constitutively expressed on T-cells, B-cells, myeloid dendritic cells and tissue macrophages. PD-L1 is encoded by the *CD274/PD-L1* gene, located on chromosome 9p24.1, in close proximity to *PDCD1LG2* (programmed cell death ligand 2). PD-L1 can be upregulated in tumor cells and in a number of different tissue types in response to INF-γ and other inflammatory mediators [4]. Its receptor PD-1 is an immune checkpoint protein, expressed on the surface of several immune cells, particularly cytotoxic T-cells [4-6]. Tumor immune escape can occur through several potential mechanisms, mediated by high tumor expression of PD-L1 and/or tumor immune infiltration by PD-1-positive T-lymphocytes: (1) Binding of PD-L1 by PD-1 on T-cells can lead to functional anergy or apoptosis of T-cells. (2) In regulatory T-cells (Tregs), ligation of PD-1 by PD-L1 can potentially induce immunologic tolerance. (3) Reverse signaling from PD-L1 may prevent tumor cells from apoptosis and (4) PD-L1 interaction with the CD80 ligand can promote inhibition of immune response [7-13].

Aberrant PD-L1 expression has been demonstrated in many solid cancers [14], including melanoma [15, 16], lung cancer [17, 18] and colon cancer [19, 20]. Moreover, selective 9p24.1 gene amplification has recently been identified as an important mechanism for increased PD-L1 protein expression in nodular sclerosing Hodgkins lymphoma and primary mediastinal large B-cell lymphoma [21]. Subsequently, 9p24.1 gene amplification has been detected in a subgroup of gastric carcinoma, colon carcinomas, triple-negative breast cancers and glioblastomas [22, 23]. In a meta-analysis of 25 studies, Wu et al showed that overexpression of PD-L1 is associated with worse 3-year overall survival (OS) in esophageal, hepatocellular and urothelial carcinoma as well as gastric cancer whereas this association was not found in carcinomas of the lung and melanoma [14].

In HNSCC, limited data suggest that PD-L1 is expressed in tumor cells in 50-70% of the cases and that PD-1-positive tumor infiltrating lymphocytes (TIL) may be more common in cancers that are HPV positive than HPV-negative [24-30]. PD-L1 expression is found in various HNSCC tumor cell lines and upregulation was demonstrated in response to the proinflammatory cytokines INF-γ, TNF-α and IL-1β. Interestingly, in a preclinical HNSCC model system, PD-1 mediated signals on T-cells can inhibit antitumor immune response [27, 31].

To further explore the PD-1/PD-L1 axis in HNSCC, we evaluated and compared frequencies of PD-1 and PD-L1 immunohistochemical expression in a clinically well characterized cohort of OCSCCs of 80 primary tumors and 28 associated nodal metastasis and determined its prognostic impact. In addition we analyzed the *PD-L1* gene locus on 9p24.1 by fluorescence *in situ* hybridization (FISH) and identify *PD-L1* amplification as an important mechanism of PD-L1 upregulation in OCSCC.

## RESULTS

### Expression of PD-L1 and PD-1

**Figure 1:**
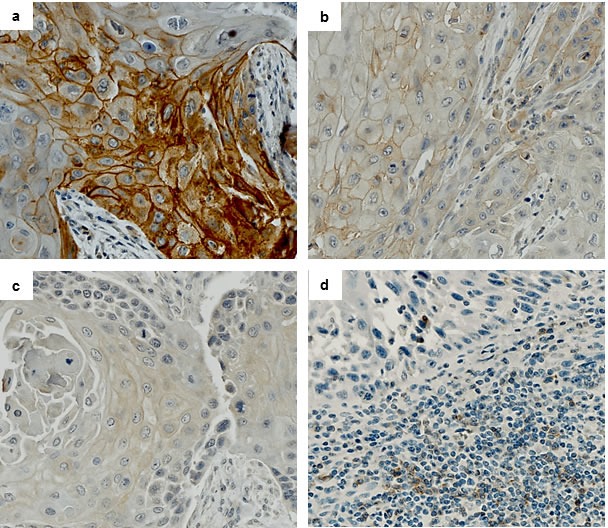
Squamous cell carcinoma of the head and neck, showing membraneous PD-L1 expression in tumor cells with **a.** strong staining (3+), **b.** intermediate staining (2+) and **c.** weak staining (1+) intensity. Fibroblasts within tumor stroma are negative. d) PD-1 staining in squamous cell carcinoma with intermingled tumor-infiltrating lymphocytes (TILs). PD-1 staining is only seen in TILs, while carcinoma cells are negative. (**a.**-**c.** PD-L1 immunohistochemistry, **d.** PD-1 immunohistochemistry).

**Figure 2:**
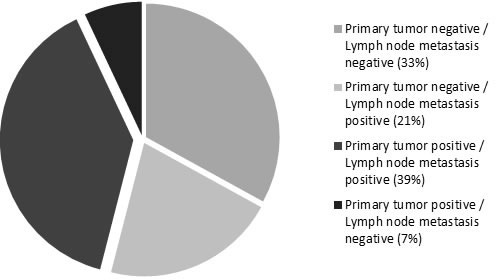
Concordance and discordance of PD-L1 expression in primary tumor and matched lymph node metastases

PD-L1 expression was evaluated in tumor cells of primary oral cavity squamous cell carcinoma and associated nodal metastasis by immunohistochemistry. PD-L1 expression in at least 5% of tumor cells was found in 36/80 (45%) of OCSCCs. Mean percentage of positive tumor cells (any strength of expression) was 60% (range: 15%-90%). Strong, membranous staining (3+) was found in 13/36 (36%) cases, intermediate staining (2+) in 13/36 (36%) cases, whereas 10/36 (28%) cases demonstrated weak staining (1+) (Figure 1a-1c). In 28 of the 80 analyzed cases, primary tumors and corresponding nodal metastases were available for assessment. Evaluation of the immunohistochemical PD-L1 staining results in these 28 cases was performed both on tissue cores as well as whole tissue slides to exclude confounding effects due to possible tissue heterogeneity. Among 13 primary tumors positive for PD-L1 expression, 11/13 (85%) of corresponding nodal metastases showed concomitant positive expression of PD-L1, whereas 2/13 (15%) cases displayed loss of staining in the metastasis. A discrepancy between staining results in tissue cores and whole slides was only seen in one case, which showed positive PD-L1 staining in the whole tissue section and negative staining in the tissue cores. In 15 primary tumors negative for PD-L1 expression, 9/15 (60%) of matching nodal metastases were PD-L1 negative, whereas 6/15 (40%) cases were PD-L1 positive. Altogether, in 20/28 (72%) cases, concordant PD-L1 expression was found in matched primary tumors and nodal metastases, and in 8/28 (28%) cases a discordance of PD-L1 expression was present in primary tumor specimen and corresponding nodal metastases (Figure 2).

We next evaluated PD-1 expression in both neoplastic as well as inflammatory cells by immunohistochemistry. 79 cases were available for IHC. PD-1 was not expressed in tumor cells whereas tumor-infiltrating lymphocytes (TILs) showed PD-1 staining. 41/79 (52%) cases contained PD-1 positive TILs. Mean percentage of PD-1 positive TILs was 6% (range 3%-20%). 25/36 (69%) PD-L1 positive carcinomas and 16/43 (37%) of PD-L1 negative carcinomas contained PD-1 positive TILs (Figure 1d). There was no significant correlation of PD-L1 and PD-1 expression.

### Evaluation of *CD274/PD-L1* copy number status by fluorescence *in situ* hybridization

**Figure 3:**
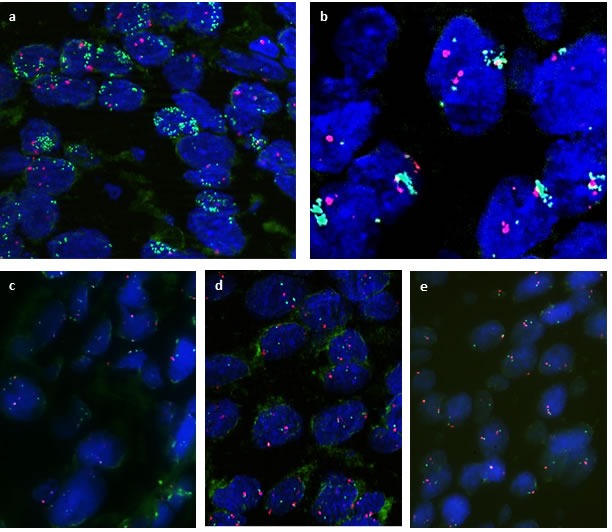
Fluorescence *in situ*-hybridization (FISH) for *PD-L1* *PD-L1* gene is labelled in green, centromer 9 in red. FISH analysis showing **a.**, **b.** high level amplification, with *PD-L1/CEP9* ratio ≥ 4, indicated by clusters of green fluorochrome labeling *PD-L1* **c.** low level amplification, *PD-L1/CEP9* ratio ≥ 2.0 - < 4; **d.** Polysomy of *PD-L1* gene locus; **e.** *PD-L1* disomy. (**a.**-**d.** SPEC *CD274, PDCD1LG2/CEN*9 Dual Color Probe).

Given the broad range of PD-L1 immunohistochemical expression in OCSCC, which could not be easily explained by autocrine or paracrine upregulation, we further explored the copy number status of the *CD274/PD-L1* gene locus by FISH. *PD-L1* FISH analysis was performed in all 80 primary tumors and in 16 corresponding lymph node metastases. 15/80 (19%) primary tumors fulfilled the criteria for amplification: in 12/80 cases (15%), high level amplification of the *PD-L1* gene locus, as defined by *PD-L1/CEP9* ratio ≥ 4.0 was seen (Figure 3a, 3b) and 3/80 (4%) cases showed low-level *PDL-1* amplification as defined by *PD-L1/CEP9* ratio ≥ 2.0 and ≤ 4.0 (Figure 3c). Gene copy number gain was restricted to tumor cells and was not present in the inflammatory cell component. A polysomy defined as average *PD-L1* copy number > 3 signals/cell was seen in 16/80 (20%) cases (Figure 3d). 49/80 (61%) cases were disomic for the *PD-L1* gene locus at 9.24.1 (Figure 3e). The *PD-L1* copy number status was concordant in the primary tumor and corresponding lymph node metastases.

### Correlation between *PD-L1* gene amplification and *PD-L1* copy number gain with PD-L1 protein expression

Results for both PD-L1 immunohistochemistry and *CD274/PD-L1* FISH were available for all 80 cases (Table 2 and Supplementary Table 1). Tumors with *CD274/PD-L1* gene amplification displayed PD-L1 positivity by immunohistochemistry in only 11/15 (73%) cases. In contrast, 25/65 (38%) cases without *CD274/PD-L1* amplification were PD-L1 immunopositive. Cases with *CD274/PD-L1* amplification were PD-L1 immunopositive in a significantly higher frequency than cases without amplification (*p* = 0.01). 7/13 carcinomas with strong, membranous PD-L1 immunostaining (score 3+) showed high or low level *CD274/PD-L1* amplification, 3/13 showed a polysomy and 3 cases displayed a normal copy number status. Interestingly, two of the highly amplified carcinomas and two of the tumors with low-level *CD274/PD-L1* amplification were PD-L1 immunonegative (score 0). To confirm the findings and to exclude false negative staining due to tumor heterogeneity in these 4 cases, FISH analysis and immunohistochemistry were repeated on whole tissue sections, giving identical results. Altogether, 40/80 (50%) cases were negative for both PD-L1 immunohistochemistry and *PD-L1* FISH.

**Table 1:** Clinicopathological data in relation to PD-L1 immunohistochemical expression

Clinicopathologic Parameters	PD-L1 positive	PD-L1 negative
n (%)	36 (45%)	44 (55%)
Median age (years) Age range (years)	57 38-86	60 44-84
Gender Male Female	23 (64%) 13 (36%)	31 (70%) 13 (30%)
Differentiation G1 G2 G3	1 (3%) 29 (80%) 6 (17%)	3 (7%) 27 (61%) 14 (32%)
Tumor Size T1 T2 T3 T4a/b	4 (11%) 15 (42%) 5 (14%) 12 (33%)	8 (18%) 17 (39%) 9 (20%) 10 (23%)
Lymph node metastasis Positive Negative	26 (72%) 10 (28%)	19 (43%) 25 (57%)
HPV (Type 16) Positive Negative	1 (3%) 35 (97%)	4 (9%) 40 (91%)

**Table 2:** *CD274/PD-L1* FISH and PD-L1 immunohistochemistry

*CD274/PD-L1* FISH	Cases	PD-L1 IHC			
	*n* = 80	Score 3+	Score 2+	Score 1+	Score 0
High Level Amplification	12 (15%)	7/12	1/12	2/12	2/12
Low Level Amplification	3 (4%)	1/3	0/3	0/3	2/3
Polysomy	16 (20%)	3/16	3/16	3/16	7/16
Disomy	49 (61%)	3/49	8/49	5/49	33/49

### Correlation of PD-1, PD-L1 expression and *CD274/PD-L1* amplification in tumor tissue with clinicopathologic features

All features of clinicopathological data in relation to PD-L1 immunohistochemical expression are summarized in Table 1. In 5 cases, HPV DNA was detected, indicating HPV associated carcinogenesis, with one of these cases showing *CD274/PD-L1* high level amplification and PD-L1 immunopositivity. The remaining four cases did not express PD-L1. Patients with PD-L1 immunopositive tumors had a significantly higher risk for lymph node metastasis at diagnosis (*p*-value = 0.009), whereas PD-L1 immunohistochemical expression was not relevantly correlated with age, gender, grading, tumor size or HPV-positivity. In contrast, both *CD274/PD-L1* copy number status and PD-1 immunohistochemical expression were found to be not relevantly correlated with the presence of nodal metastases and were also not relevantly correlated with age, gender, grading, tumor size, or HPV-positivity.

### Correlation of PD-L1 expression with clinical data

**Figure 4:**
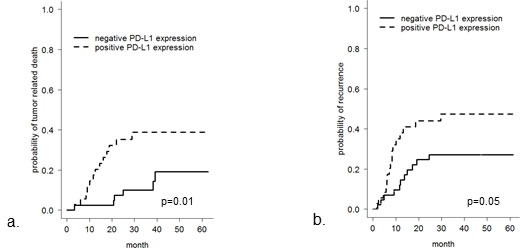
Overall tumor-related survival and recurrence-free survival in patients with SCC of the oral cavity with respect to PD-L1 immunohistochemical status Overall tumor-related survival **a.** and recurrence-free survival **b.** is worse in SCC patients with tumoral PD-L1 immunopositivity (*p* = 0.01 and 0.05, respectively).

In the SCC patient cohort with PD-L1 immunopositive tumors, the risk of tumor related death was significantly increased (*p* = 0.01) (Figure 4a) which was also the case for the risk of recurrence (*p* = 0.05) (Figure 4b). In contrast, PD-1 expression, *CD274/PD-L1* copy number, pT category, presence of lymph node metastases or tumor grading were not relevantly associated with these risks (data not shown).

## DISCUSSION

Malignant tumors can escape the host´s immune system by a process termed “immunoediting”, turning the immune microenvironment into an immunosuppressive state. An important pathway in the immune anti-tumor response is the PD-1/PD-L1 axis [10, 32, 33]. PD-L1 expression in tumor cells is thought to be predictive for tumor response to immunomodulatory therapies targeting the PD-1/PD-L1 pathway [34]. Data on PD-1 and PD-L1 expression in HNSCC concerning prevalence, prognostic impact and variation of expression in disease course is limited.

In the present study, we provide novel molecular genetic evidence that the *CD274/PD-L1* gene is amplified in OCSCC and that gene amplification is accompanied by immunohistochemical PD-L1 protein overexpression in only a subset of amplified cases. We also demonstrate that the PD-L1 protein expression status in primary tumors and corresponding nodal metastasis is discordant in a significant number of cases investigated.

Using immunohistochemistry and a cut-off value for positivity defined as at least 5% tumor cells displaying membranous PD-L1 staining, we found PD-L1 expression in 36/80 (45%) of OCSCC, a range similar to published data. To date, only few studies have evaluated PD-L1 expression in OCSCC [27] [31] [24] [25] [28] with positivity rates ranging between 46%-87%. These variable positivity rates are likely due to several factors: (1) Use of different cut-off values for definition of positivity; for example, a study by Ukpo et al used a cut-off value of 5%, whereas Badoual et al and Strome et al defined positivity as any staining > 0% [27] [24] [28]; (2) cytoplasmic staining, in addition to membranous staining was counted as positive by some authors; (3) use of different antibodies clones for immunohistochemistry [27] [31] [24] [25] [28] and (4) inclusion of a high percentage of HPV-positive cases in one of the studies [28].

Up to date, there is limited data concerning changes of PD-L1 expression in tumors during metastatic progression and none of the studies up to date have evaluated SCCs of the head and neck. Results of our own study show that in only 72% of cases, the PD-L1-status was retained in nodal metastatic lesions. In two studies comparing matched tumor samples with corresponding metastatic lymph nodes involving patients with RCC and triple-negative breast cancer, respectively, the concordance rates were considerably higher [33, 35]. Tissue heterogeneity of PD-L1 protein expression or change of PD-L1 expression during metastatic disease progression might contribute to low concordance rates, but further functional follow up studies are needed to clarify this issue.

Tolerance of PD-L1 positive tumors by the immune system is mediated by interaction of PD-L1 on tumor cells with its receptor PD-1, preferentially expressed on Tregs and other immune cells. Upon activation of PD-1/PD-L1, the anti-tumor response of the immune system is attenuated as T-cell functions are suppressed by the mechanisms named above. In HNSCC, co-receptor signals on T-cells mediated by PD-1 can inhibit antitumor response in a pre-clinical model [8, 27, 33, 36, 37]. In our study cohort, all primary tumors were PD-1 negative, whereas PD-1 was expressed in TILs in 24/36 (67%) PD-L1 positive carcinomas and 16/43 (37%) of PD-L1 negative. Absolute numbers of positive TILs were low in comparison to previous studies [9], [26] and may reflect differences between the pathogenesis of OCSCC and other anatomical regions of the head and neck like the oropharynx, where HPV induced carcinogenesis is more prevalent.

In our OCSCC patient cohort, PD-L1 positive carcinomas had a significantly higher risk for nodal metastasis at the time of diagnosis (p-value 0.01). Moreover, the risk of disease-and overall tumor-related death was significantly higher in PD-L1 positive OCSCC. In comparison, results of previous studies have been contradicting in this regard, probably reflecting differences in methodologies used to assess PD-L1 status and differences in patient characteristics, including percentage of HPV-related SCC tumor subgroups [25] [28]. It is well known that human papillomavirus (HPV)-associated HNSCCs tend to have improved clinical outcomes when compared to tobacco-related head and neck cancers. In contrast to oropharyngeal cancer, where HPV has been recognized as an important causative factor for tumor development, its influence on OCSCC pathogenesis is less clear and prevalence rates are much lower.

Expression of PD-L1 in tumors cells is mediated by different mechanisms. It can be induced by autocrine or paracrine factors within the tumor microenvironment, especially INF-γ or HIF1α. In this case, PD-L1 expression is dynamic, with variable, time-dependent expression. On the other hand, expression can be driven by gene amplification events involving the 9p24.1 locus. The 9p24.1 chromosomal locus contains the *CD274/PD-L1*, *PD-L2* and *JAK2* genes [21, 23]. Selective 9p24.1 amplification has been recently recognized as an important mechanism for increased PD-L1 expression in nodular sclerosing Hodgkins lymphoma and primary mediastinal large B-cell lymphoma [21] and has also been demonstrated in gastric carcinoma, colon carcinomas, triple-negative breast cancers and glioblastomas [22, 23]. Other signaling pathways, which have been linked to oncogene-induced PD-L1 expression are, amongst others, loss of PTEN and dysregulation of the JAK/STAT pathway, the latter probably providing an additional “enhancer loop” for PD-L1 overexpression when JAK2 is amplified [16, 36, 38-40].

Using FISH analysis, we found amplification of 9p24.1, including the *CD274/PD-L1* gene locus, in 19% of OCSCC cases. The amplification driven PD-L1 expression in a subgroup of OCSCC may identify a new subgroup of HNSCC with a disease-specific genetic alteration. Further studies are needed to evaluate the impact of PD-L1 amplification on pathogenesis and disease progression and on prognosis of this newly identified subgroup of HNSCC.

Blocking the PD-1/PD-L1 axis is a promising treatment option in multiple tumor types. Up to date, several clinical studies have been conducted, including especially patients with melanoma and non-small cell lung cancer (NSCLC), but also HNSCC (reviewed in [3]). These studies have demonstrated better response rates in patients with high PD-L1 expression. In addition, immune checkpoint blockage has shown remarkable response rates in lymphomas, especially Hodgkins lymphoma, with *PD-L1* amplification [41]. Response to anti-PD-1 treatment was also found, in a significantly lower percentage, in PD-L1 negative cases [8, 33, 42-45]. Considering the fact that the interaction of PD-L1 on tumor cells with PD-1 on TILs leads to escape of cancer cells from the immune system, it is not surprising, that upregulation of PD-1 by recombinant cytokines, stimulatory antibodies or transfer of activated adoptive T-cells increases the effect of PD-1/PD-L1 pathway blockage [27, 46, 47]. The identification of *CD274/PD-L1* gene copy number gain as a potential mechanism for PD-L1 overexpression in the present study may provide a rationale for treatment of HNSCC patients, especially in a subgroup of OCSCC with *PD-L1* amplification. Future studies have to investigate whether *PD-L1* copy number status, which is stable in the primary tumor and corresponding lymph node metastasis, might be a better predictive marker for tumor response to blockage of PD-1/PD-L1 pathway [34] than PD-L1 immunohistochemical status. In any case, the correlation between *CD274/PD-L1* gene amplification and the PD-L1 immunohistochemical status is surprisingly poor, with 4 amplified cases being PD-L1 immunonegative. The reason for this discrepancy is currently not clear, but may involve technical problems with the antibody clone used or posttranscriptional or posttranslational modifications.

In summary, we show that PD-L1 is expressed in 45% of OCSCC, when a 5% cut-off of positive cells is applied. PD-L1 positive cases have a significantly higher risk for nodal metastasis at diagnosis. PD-L1 expression is associated with higher risk for overall tumor-related death and recurrence. Although PD-L1 expression status in corresponding nodal metastasis is equivalent to expression in primary tumors in the majority of cases, a considerable number of cases (32%) were discordant. *CD274/PD-L1* amplification is a novel finding in OCSCC and is a frequent event, often (but not always) associated with high PD-L1 expression levels and stable during metastatic progression. Based on these data, we propose to include *CD274/PD-L1* amplification analyses into studies recruiting patients for immune checkpoint protocols.

## MATERIALS AND METHODS

### Patient cohort

The retrospective cohort consisted of 80 patients, who underwent surgical resection of squamous cell carcinoma of the oral cavity between 2008 and 2010 at the Klinikum rechts der Isar of the Technical University of Munich, Germany. H&E stained sections were reviewed by three pathologists (MS, ED, KS). Grading and staging was undertaken according to the current World Health Organization Classification of Tumors of Head and Neck (2004) and the AJCC tumor, node, metastasis (TNM) classification (7^th^ edition). Complete clinicopathologic data including age, gender, localization of primary tumor, local, nodal and distant recurrences and overall and disease-free survival were available for all patients (Supplementary Table 1). All patients underwent primary resection of tumors with curative intent following a standardized surgical procedure [48]. Patients did not have distant metastasis at diagnosis. Patients with lymph node metastasis at diagnosis received adjuvant radiotherapy. Mean follow-up was 31 months (range 2 - 63 months). Approval for the study was obtained from the Ethics Review Committee of the Technical University of Munich.

### Tissue microarray construction

Formalin-fixed paraffin-embedded tumor samples were assembled into a tissue microarray (TMA) using a Tissue Microarrayer (Beecher Instruments, Sun Praierie, USA) with a core size of 0,6 mm. A minimum of 2 and (where feasible) up to 4 tumor cores from tumor invasion front and tumor center were taken from the primary tumors in areas previously marked by two pathologists (MS, ED). Corresponding regional nodal metastasis from 28 patients were also included in the TMA, selecting 1 or (where feasible) 2 different areas from the metastatic deposits, depending on size of metastasis.

### Immunohistochemistry

IHC was performed on 2 μm sections from each TMA using a PD-L1 primary antibody (Cell Signaling, clone E1L3N) at a dilution of 1:100 and a PD-1 primary antibody (Cell Marque, clone 11RQ-22) at a dilution of 1:50. Stainings were run on an automated immunostainer with an iVIEW DAB detection kit (Ventana Medical Systems, Roche, Mannheim, Germany). Immunohistochemical expression of PD-L1 in tumor cells was assessed by counting the percentage of positive tumor cells and quantifying staining intensity in a 4-tiered grading system including “no staining” (0), “weak staining” (1+), “intermediate staining” (2+), “strong staining” (3+). PD-L1 positivity was arbitrarily defined as at least 5% tumor cells displaying membranous PD-L1 staining of any intensity. The cut-off value of 5% was chosen, because many clinical trials involving immunomodulatory therapeutics have chosen this value. Immunohistochemical expression of PD-1 in TILs was assessed by counting the percentage of PD-1 positive TILs. PD-1 positivity was counted as at least 1% positive TILs.

### *PD-L1* fluorescence *in situ* hybridization

Dual-color FISH analysis was performed on 2μm sections from formalin-fixed paraffin-embedded tissue. Sections were deparaffinized, dehydrated in 100% ethanol and air-dried. For *in situ* hybridization, the SPEC *CD274, PDCD1LG2/CEN*9 Dual Color Probe containing a mixture of fluorochrome direct labelled *SPEC CD274, PDCD1LG2* probe specific for the *CDC274/PD-L1* and *PDCD1LG2/PD-L2* genes located at 9p24.1 and an orange fluorochrome labeled *CEN 9* probe specific for the classical satellite III region of chromosome 9 (D9Z3) at 9q12 (Zytovision, Bremerhaven, Germany) was used according to the manufacturer´s instructions. At least 20 nuclei per sample were counted. *PD-L1* amplification was defined as *PD-L1/CEP9* ratio ≥ 2.0, with high level amplification defined as ≥ 4.0 and low level amplification defined as ≥ 2.0 and ≤ 4.0. Polysomy 9 was defined as average *PD-L1* copy number > 3 signals/cell.

### HPV analysis

DNA was isolated from formalin-fixed, paraffin-embedded tissue of primary tumor samples using the QIAamp DNA FFPE Tissue Kit (Qiagen, Hilden, Germany) in combination with the QiaCube (Qiagen, Hilden, Germany) according to the supplier's instructions. HPV analysis and determination of HPV subtypes was performed with the HPV LCD 3.5 Array - Kit (Chipron GmbH, Berlin, Germany) according to the manufacturer's recommendations.

### Statistics

Descriptive and exploratory statistical analyses were performed using SPSS 21 (SPSS Inc, Chicago, IL, USA) and R 3.2.0 (R Foundation for Statistical Computing, Vienna, Austria). The distribution of qualitative data was compared between groups using χ^2^-test or Fisher's exact test, depending on the cell counts of corresponding contingency tables. Likewise, quantitative data was compared between groups using the non-parametric Mann-Whitney *U* test. Cumulative incidence functions were estimated for censored outcomes to account for competing risks such as death. All statistical tests were performed on exploratory two-sided 5% significance levels.

## SUPPLEMENTARY MATERIAL TABLE


