# Supplementary material for: CD274/PD-L1 gene amplification and PD-L1 protein expression are common events in squamous cell carcinoma of the oral cavity
Source: Oncotarget. 2016 Feb 22;7(11):12024–34. doi: 10.18632/oncotarget.7593 (PMC4914266; doi:10.18632/oncotarget.7593)
Supplement: Supplementary file 1 [file oncotarget-07-12024-s001.pdf]

CD274/PD-L1 gene amplification and pd-l1 protein expression are common events in squamous cell carcinoma of the oral cavity

Supplementary Material

Case	PD-L1 IHC	PD-L1 IHC	PD-1 IHC	HPV	PD-L1 FISH	Gender	Age	*T*	*N*	Grading	Recurrence	Local Recurrence	Nodal Recurrence	Distant Reccurence	Recurrence (Localisation not available)	Overall Survival
	Staining Intensity	Percentage Positivity	Percentage Positivity	1=positive<br>0=negative	Amplification<br>3=high level<br>2=low level	1=male<br>2=female					1=yes<br>0=no	1=yes<br>0=no	1=yes<br>0=no	1=yes<br>0=no	1=yes<br>0=no	1=dead<br>0=alive
					1=Polysomy<br>0=Disomy											
1	0		0%	0	0	1	70,1	*2*	*0*	3	0	0	0	0	0	0
2	0		0%	0	0	2	44,1	*1*	*0*	2	0	0	0	0	0	0
3	0		10%	0	0	1	49,4	*3*	*0*	3	0	0	0	0	0	0
4	0		0%	0	0	2	54,9	*2*	*2b*	3	0	0	0	0	0	0
5	0		0%	0	0	1	59,9	*4a*	*2b*	2	0	0	0	0	0	1
6	0		3%	0	2	1	60,8	*2*	*0*	1	1	1	0	0	0	0
7	0		0%	1	0	1	63,6	*4a*	*0*	2	1	0	0	0	1	1
8	3+	80%	20%	0	1	2	85,8	*2*	*1*	2	0	0	0	0	0	1
9	1+	30%	0%	0	0	1	70,3	*4*	*2c*	2	1	0	0	1	0	1
10	0		0%	0	1	1	68,8	*1*	*0*	2	0	0	0	0	0	1
11	2+	30%	5%	0	0	1	62,4	*2*	*1*	2	0	0	0	0	0	1
12	0		0%	0	0	2	69,7	*4a*	*0*	3	0	0	0	0	0	0
13	0		0%	0	3	2	67,7	*4a*	*0*	2	1	0	0	1	0	1
14	2+	15%	5%	0	0	1	49,4	*2*	*0*	2	1	1	1	0	0	1
15	1+	20%	3%	0	0	2	49,9	*2*	*0*	2	0	0	0	0	0	0
16	0		0%	0	0	1	48,5	*2*	*1*	2	0	0	0	0	0	0
17	0		5%	0	1	1	60,1	*3*	*2b*	2	0	0	0	0	0	0
18	0		0%	0	0	1	52,7	*1*	*0*	2	0	0	0	0	0	0
19	2+	60%	0%	0	0	2	71,8	*2*	*2b*	2	0	0	0	0	0	0
20	0		0%	0	3	2	75	4a	2b	2	1	1	1	0	0	0

21	0		5%	0	0	1	52	2	2c	3	1	0	1	0	0	0
22	0		0%	0	1	1	56,2	2	0	2	0	0	0	0	0	0
23	3+	80%	3%	0	1	2	41,0	1	1	2	0	0	0	0	0	0
24	0		0%	0	0	1	72,5	3	1	1	0	0	0	0	0	1
25	0		0%	0	0	2	63,4	1	0	2	0	0	0	0	0	0
26	1+	70%	3%	0	0	1	75,0	3	0	2	1	0	1	0	0	1
27	0		3%	0	0	2	56,2	2	2b	2	1	1	0	0	0	1
28	0		0%	0	0	2	56	2	1	2	1	0	0	1	0	0
29	1+	50%	3%	0	1	1	57,1	2	2b	2	0	0	0	0	0	0
30	0		5%	0	1	1	47,9	1	0	2	0	0	0	0	0	0
31	0		3%	0	1	1	60,1	1	0	2	0	0	0	0	0	0
32	0		0%	0	0	1	62,2	2	2c	2	0	0	0	0	0	1
33	3+	30%	3%	0	3	1	44,1	4a	2c	3	0	0	0	0	0	0
34	2+	30%	5%	0	0	1	57,9	2	1	2	0	1	0	0	0	0
35	0		10%	0	0	2	71,9	2	1	2	1	1	0	0	0	1
36	0		3%	0	0	1	53	2	2b	3	1	0	1	1	0	1
37	0		0%	1	0	2	55,7	3	0	3	0	0	0	0	0	1
38	2+	30%	0%	0	3	1	56,9	4a	0	2	0	0	0	0	0	0
39	2+	80%	0%	0	1	1	80	3	1	2	1	1	1	0	0	1
40	3+	40%	3%	0	1	1	54,6	4a	2c	2	1	0	1	0	0	1
41	1+	70%	0%	0	0	2	56,0	2	0	3	1	0	1	0	0	0
42	0		3%	0	0	2	83,6	3	0	2	0	0	0	0	0	1
43	0		3%	0	0	1	62,4	2	0	2	0	0	0	0	0	0
44	0		0%	0	0	1	48,1	4a	0	3	0	0	0	0	0	0
45	0		0%	0	0	1	47,4	2	0	3	1	1	0	0	0	0
46	0		3%	0	1	2	68,3	2	0	2	1	1	1	1	0	1
47	1+	50%	3%	0	1	1	53,9	4a	2b	2	1	1	1	0	0	1
48	0		0%	0	0	1	51,1	3	0	3	1	0	1	1	0	1
49	3+	90%	10%	0	0	1	52,2	1	2b	2	0	0	0	0	0	0

50	3+	50%	0%	0	0	1	53,2	*4a*	*2c*	3	1	0	0	1	0	1
51	0		0%	0	0	1	66,3	*2*	*0*	2	0	0	0	0	0	0
52	3+	60%	0%	0	3	2	45,6	*3*	*2b*	3	1	0	1	1	0	1
53	0		0%	0	0	1	61,7	*4a*	*2c*	3	0	0	0	0	0	1
54	0		0%	0	0	2	67,3	*2*	*0*	3	0	0	0	0	0	0
55	2+	40%	20%	0	0	1	47,6	*2*	*1*	2	0	0	0	0	0	0
56	2+	20%	0%	0	0	1	68,6	*1*	*2b*	2	1	1	0	0	0	1
57	0		0%	0	0	1	65,0	*3*	*1*	2	0	0	0	1	0	1
58	0		n.a	0	0	1	59,5	*1*	*1*	2	0	0	0	0	0	0
59	3+	70%	3%	0	3	2	58	4a	2b	2	1	0	1	0	0	0
60	0		3%	0	0	1	62,6	*4a*	*2c*	2	0	0	0	0	0	0
61	3+	60%	3%	0	3	1	54,2	*4a*	*2a*	2	0	0	0	0	0	0
62	2+	80%	3%	0	0	2	76	2	2b	3	1	1	0	0	0	0
63	3+	80%	0%	0	0	2	52,7	*4a*	*2c*	2	0	1	1	0	0	0
64	0		0%	0	1	1	71,4	*3*	*2b*	3	0	0	0	0	0	0
65	0		5%	1	0	1	44,3	*3*	*0*	2	0	0	0	0	0	0
66	1+	80%	5%	0	0	1	45,7	*3*	*0*	2	1	1	0	0	0	1
67	2+	60%	0%	0	1	2	49,9	*2*	*2b*	2	1	1	1	0	0	1
68	3+	50%	3%	0	3	2	69,2	*4a*	*2b*	2	0	0	0	0	0	0
69	0		10%	0	0	1	53,5	*4b*	*2b*	3	0	0	0	0	0	0
70	3+	50%	0%	0	2	2	72,0	*3*	*0*	1	0	0	0	0	0	0
71	0		0%	1	0	1	55,4	*4a*	*1*	2	0	0	0	0	0	1
72	2+	60%	3%	0	0	2	57,5	*2*	*2b*	2	0	0	0	0	0	0
73	1+	60%	3%	1	3	1	64,9	*2*	*2b*	2	1	1	1	1	0	1
74	1+	70%	20%	0	1	1	70,2	*4a*	*0*	3	0	0	0	0	0	0
75	3+	50%	3%	0	3	1	37,9	*1*	*0*	2	0	0	0	0	0	0
76	0		0%	0	0	1	60,6	*1*	*0*	1	0	0	0	0	0	0
77	2+	60%	3%	0	0	1	53,1	*4a*	*2c*	2	0	0	0	0	0	1
78	2+	60%	3%	0	3	1	66,6	*2*	*0*	2	0	0	0	0	0	0

79	1+	30%	20%	0	3	1	42,4	*2*	*2c*	2	1	1	1	0	0	1
80	0		0%	0	2	1	66,8	*2*	*0*	2	0	0	0	0	0	0
